# Linkage of catalysis and 5′ end recognition in ribonuclease RNase J

**DOI:** 10.1093/nar/gkv732

**Published:** 2015-08-07

**Authors:** Xue-Yuan Pei, Patricia Bralley, George H. Jones, Ben F. Luisi

**Affiliations:** 1 Department of Biochemistry, University of Cambridge, Tennis Court Road, Cambridge CB2 1GA, UK; 2 Department of Biology, Emory University, Atlanta Georgia, USA

## Abstract

In diverse bacterial species, the turnover and processing of many RNAs is mediated by the ribonuclease RNase J, a member of the widely occurring metallo-β-lactamase enzyme family. We present crystal structures of *Streptomyces coelicolor* RNase J with bound RNA in pre- and post-cleavage states, at 2.27 Å and 2.80 Å resolution, respectively. These structures reveal snapshots of the enzyme cleaving substrate directionally and sequentially from the 5′ terminus. In the pre-cleavage state, a water molecule is coordinated to a zinc ion pair in the active site but is imperfectly oriented to launch a nucleophilic attack on the phosphate backbone. A conformational switch is envisaged that enables the in-line positioning of the attacking water and may be facilitated by magnesium ions. Adjacent to the scissile bond, four bases are stacked in a tightly sandwiching pocket, and mutagenesis results indicate that this organization helps to drive processive exo-ribonucleolytic cleavage. Like its numerous homologues, *S. coelicolor* RNase J can also cleave some RNA internally, and the structural data suggest how the preference for exo- versus endo-cleavage mode is linked with recognition of the chemical status of the substrate's 5′ end.

## INTRODUCTION

The ribonuclease RNase J is a key enzyme in RNA turnover and precursor processing in the model Gram-positive bacterium *Bacillus subtilis* and numerous other bacterial and archaeal species (1–9). The enzyme appears to have had an ancient evolutionary origin, because homologues of RNase J have been identified in genomes of roughly half the bacterial species characterized to date; moreover, the enzyme's structure demonstrates membership with an extensive and widely distributed structural family of Zn-dependent metallo-β-lactamases (1). The family takes its name from the founding members that cleave β-lactam rings hydrolytically, and all metallo-β-lactamases bear two zinc ions in a catalytic site that is proposed to activate water to become a potent nucleophile. Members of the family include the bacterial endoribonuclease RNase Z that matures the 3′ end of tRNAs (10), as well as enzymes involved in mRNA cleavage and the deadenylation enzymes of metazoans and fungi (1).

The β-lactamase domain present in all members of metallo-β-lactamases, consists of a central core of two β-sheets flanked by α-helices, with the active site zinc ions located at the edge of the two β-sheets (6–9). The members of the ribonuclease sub-group of the metallo-β-lactamase family often have auxiliary domains that contribute to substrate recruitment. Thus, in addition to the eponymous β-lactamase fold, RNase J bears C-terminal and β-CASP domains (1). Crystal structures of RNase J from *B. subtilis*, *Thermus thermophilus* and *Deinococcus radiodurans* reveal that the catalytic site is situated in a deep inter-domain cleft between the β-lactamase core and the β-CASP domain (6–9).

RNase J can cleave mRNA by either endonucleolytic or exonucleolytic mode, with preference depending on the 5′ end of the RNA (2–4). *B. subtilis* RNase J prefers to cleave substrates in an exo-nucleolytic mode if these carry a 5′-monophosphate or hydroxyl group (2–4). The discrimination of the 5′ end has been proposed to arise from the optimal spacing between a 5′ phosphate binding pocket and the active site (8). Two isoforms of RNase J, known as RNase J1 and RNase J2, have been identified in *B. subtilis*, *Staphylococcus aureus* and *Streptococcus mutans*, and these have been functionally characterized (3,11,12). The *B. subtilis* enzymes have been shown to form stable heterodimers, and the RNase J2 subunits of *B. subtilis* and *Staphylococcus aureus* have endonucleolytic activity but almost no exo- activity, and may play a structural role in stabilizing RNase J1 in the heterodimer (3,11).

Recently, a homologue of RNase J has been identified in the representative actinobacterium, *Streptomyces coelicolor* (SCO5745) (13) (Supplementary Figure S1). Only one gene for the enzyme is encoded in the *S. coelicolor* genome, indicating that the protein does not form a hetero-oligomer resembling the RNase J1/RNase J2 complex of *B. subtilis*. Here we present the crystal structure of *S. coelicolor* RNase J in a catalytically stalled state. The structure reveals a homotetrameric quaternary organization that unexpectedly carries intact nucleotides of RNA at each of the active sites. This pre-cleavage structure, solved at 2.27 Å resolution, provides details of the interactions of the RNA with the catalytic site, including a network of water molecules which may facilitate acid-base chemistry. The enzyme appears to be stalled with bound substrate because a metal cofactor such as Mg^++^ may be required to approach the geometry required to attain the transition state. We also present the structure of the post-cleavage complex at 2.80 Å resolution, prepared by soaking Mn^++^ in the co-crystal of the RNase J/RNA complex. Comparison of the pre- and post-cleavage states reveals conformational changes that follow hydrolytic cleavage.

Like the *B. subtilis* and *D. radiodurans* homologues, the *S. coelicolor* RNase J has preference for either endo- or exo-ribonuclease mode depending on the chemical status of the 5′ end of the substrate. We identify mutations that affect this preference and propose a model that explains how recognition of the 5′ end of the substrate is coupled with organization of the active site to define the cleavage mode.

## MATERIALS AND METHODS

### RNase J expression and purification

*Streptomyces coelicolor* gene *sco5745* encoding RNase J was cloned into the T7 expression vector pET19b as an NdeI / BamHI fragment to generate plasmid pJSE2201 encoding RNase J residues 1–561 (13). *Escherichia coli* Rosetta2(DE3)pLysS transformed with pJSE2201 was grown at 37°C and induced at 18ºC and OD_600_ nm between 0.35 and 0.4 by 0.2 mM isopropyl-β-D-thiogalactoside. Growth was continued overnight at 18°C and the cells were harvested at OD_600_nm 1.9 and lysed with an emulsiflex device (Amestin) at 10 Kpsi in buffer composed of 50 mM Tris-HCl pH 7.5, 500 mM NaCl, 20 mM imidazole, 5% v/v glycerol, 4 mM β-mercaptoethanol, 2 mM PMSF, 5 mM TCEP and 50 units/ml DNase I. The lysate was loaded on a Ni-NTA column (GE Healthcare), and the column was washed with buffer containing 50 mM Tris-HCl pH 8.3, 500 mM NaCl, 100 mM imidazole, 5% v/v glycerol, 4 mM β-mercaptoethanol, 2 mM PMSF, 5 mM TCEP and 0.05 mM EDTA. The protein was eluted with a linear gradient from 100 mM to 250 mM imidazole, and fractions enriched in RNase J were dialyzed into a lower salt buffer containing 20 mM Tris-HCl, pH7.5, 25 mM imidazole, 0.5% v/v glycerol, 2 mM TCEP, 2 mM EDTA. The sample was applied on a monoQ column (GE Healthcare) and eluted in the above buffer with a gradient from 0 to 1 M NaCl. Enriched fractions were further purified by gel filtration chromatography using Superdex 200 with buffer composed of 20 mM Tris-Cl, pH 7.5, 140 mM NaCl, 0.5% v/v glycerol, 2 mM TCEP, 2 mM EDTA. Samples were concentrated to an absorbance of 6.7 at 280 nm prior to crystallization trials, measured using a NanoDrop spectrophotometer (Thermo scientific). The 260 nm/280 nm absorbance ratio of 1.09 indicated that the sample was contaminated by nucleic acid and the presence of RNA bound to RNase J was confirmed by the initial unbiased electron omit map (Supplementary Figure S2) and RNA extraction and mass spectrometry analysis (protocol provided in supplementary materials).

Mutants of RNase J were prepared by site-directed mutagenesis. RNase J mutants and apo-form were purified using the method as described above. Single fractions with an absorbance ratio of 260 nm/ 280 nm lower than 0.8 were used for RNA degradation assays.

### Crystallization, structure determination and refinement

Crystallization trials were carried out with Qiagen screens at 18°C, and initial crystals were optimized in combination with micro seeding. The best crystals of *S. coelicolor* RNase J were obtained in 27.5% w/v PEG 400, 0.1 M Tris-HCl, pH 8.5 made by mixing a 1:2 volume ratio of crystallization reservoir to protein solution.

X-ray data were collected at 100 K from single crystals cryo-protected with 30% w/v PEG 400. Zn absorption peak data were collected at 9664.63 eV (1.2829 Å) using beam-line IO2 at the Diamond Light Source, U.K. RNase J crystallized in space group P4_3_22 with two protomers in the asymmetric unit. Diffraction data were processed using the programs iMOSFLM (14), SCALA and TRUNCATE (CCP4 suite) (15).

The Zn-sites were found from the anomalous data using SHELXD (16) and the map calculated with the SAD phases was used to place the structure of *S. coelicolor* RNase J by molecular replacement using PHASER (17). Coordinates 3BK1 were used as a search model in which zinc and other heteroatoms were removed. The zinc sites matched the anomalous peaks calculated from the molecular replacement phases, confirming that MR had yielded a correct solution (Supplementary Figure S3A).

The starting phases were improved by density modification using PHENIX (18). The Fo–Fc difference map calculated from this model clearly showed the presence of positive peaks corresponding to an RNA molecule bound at the active site (Supplementary Figure S2) and single-stranded RNA was modelled into the site using COOT (19). The geometry of the RNA molecule in the crystal complex structure was validated using DSSR (20). The map was improved by density modification, solvent flattening and non-crystallographic 2-fold averaging. The structure was refined with the PHENIX suite with individual site and group ADP refinement coupled with simulated annealing. The final model has *R*_work_ and *R*_free_ values of 13.3% and 16.3%, respectively. Crystallographic statistics of the refinement are listed in Supplementary Table S1. The RNase J structure includes residues 2–452. Residues 453–561 are disordered and were built as a poly-alanine chain. All images were generated using the program PyMOL (http://sourceforge.net/projects/pymol).

To prepare the post-cleavage complex, crystals of the RNA/RNase J complex were treated with Mn^++^-ions using a combination of different concentrations and soaking times. X-ray data were collected from the treated crystals at Diamond Light source, and the structure was solved by molecular replacement using as a search model the protein component alone taken from the *S. coelicolor* RNase J (1 to 456)/RNA complex structure described above, after removal of RNA, water molecules and Zn-ions. The initial Fo–Fc omit map from the molecular replacement showed positive peaks corresponding to Zn-ions above 3 sigma level as well as positive peaks corresponding to cleaved RNA in one protomer at 2.5 sigma contouring, and an uncleaved RNA in the other protomer in the dimer above 3 sigma level.

### Analysis of co-purifying and co-crystallizing RNA

RNA was extracted from the protein used for crystallization and crystals from 6 crystallization drops (each drop has RNase J in 4.5 μl at 6.15 absorbance at 280 nm) using phenol/chloroform pH 4.5 following by ethanol/Na acetate precipitation at −20°C over night. RNA pellets were washed with 75% ethanol and dissolved in 7 μl water. The RNA samples were then purified using a 1.5% urea-acrylamide gel. Gel bands containing RNA were excised and RNA eluted with shaking at 400 rpm and 8°C for about 15 h in 10 ml of elution buffer containing 100 mM sodium acetate, pH 5.0, 10 mM EDTA (pH 8.0) and 0.1% SDS. RNA bound to the purified RNase J protein sample was extracted using the same procedure. The size of the extracted RNA estimated by mass spectrometry was 11 nucleotides (methods are provided in the Supplementary Data).

The extracted RNA samples were treated with calf intestinal alkaline phosphatase prior to ligation to the adaptor for amplification. The 5′-adaptor was the 64 nucleotides RydC RNA, and the 19 nucleotide E3 RNA was used as the 3′-adaptor. The extracted RNAs were ligated to the 5′-adaptor RydC, then purified using a Zymo-Kit, and then the 3′-adaptor E3 RNA was ligated to the product. Those ligation products were used for reverse transcription and cDNA library synthesis with random hexamer primers (Invitrogen, cat. No. 48190–011). A forward primer that include a T7 promoter was used to amplify DNA fragments containing RydC, RNase J-bound RNA and E3. Polymerase chain reaction (PCR) products were used directly in DNA sequencing (Department of Biochemistry Cambridge sequencing service).

### RNA processing assays

Fifteen μl reaction mixtures contained 1X New England Biolabs Restriction Buffer 3 (100 mM NaCl, 50 mM Tris-HCl, 10 mM MgCl_2_, 1 mM DTT, pH 7.9), 1.25 μg (ca. 1 μM) protein and 1 pmol (67nM) ^32^P-labeled substrate, maintaining the enzyme to substrate ratio used by Mathy *et al*. in the studies of the *B. subtilis* RNase J1/J2 heterotetramer (3). Reactions were incubated at 37°C for 60 min and terminated by the addition of 10 μl Sequencing Stop Solution (U.S. Biochemical Corp.). Products were separated on a 12% polyacrylamide gel with 7 M urea and subjected to autoradiography.

The *B. subtilis thrS* substrate was synthesized by *in vitro* transcription. Plasmid pJSE2250 was constructed by cloning into pCR2.1-TOPO a 384 base PCR fragment representing the *thr*S leader from *B. subtilis* BG267, and the plasmid was linearized with XbaI to generate the template for *in vitro* transcription. Primers for PCR were identical to those used by Luo *et al*. (21). Unlabeled transcripts were synthesized using the MegaScript T7 High Yield Transcription Kit (Ambion) following the manufacturer's protocol to produce a 384 base *thrS* transcript. For 5′-end monophosphate labeling, transcripts were treated with calf intestinal alkaline phosphatase, followed by incubation with T4 polynucleotide kinase (Promega) in the presence of [γ-32P]ATP (3000 μCi/mmol; Perkin Elmer). For 5′-end triphosphate labeling, the Megascript T7 High Yield reaction was modified by reducing the GTP concentration from 75 mM to 15 mM and including 70 μCi of [γ-32P] GTP (6000 μCi/mmol; Perkin Elmer).

## RESULTS

### *S. coelicolor* RNase J assembles as a homotetramer

**Figure 1.**
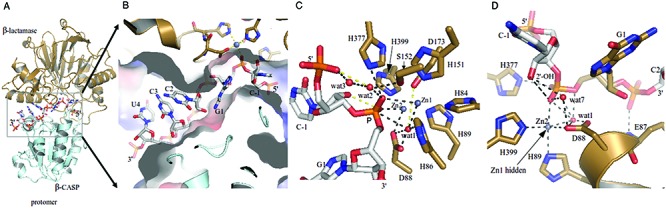
The RNA binding channel in *S. coelicolor* RNase J (SCO5745), and a sequential zoom to the active site from the structure of the pre-cleavage complex formed with RNA. (**A**) Single stranded RNA is engaged at the interface between the β-CASP and β-lactamase domains. Only one protomer of the RNase J tetramer assembly is shown for clarity. The protein and RNA are shown in cartoon and stick representations, respectively. (**B**) The RNA binding tunnel (surface representation) with bound nucleic acid (in stick representation). Some amino acids in the active site are shown in stick representation. The surface is colored blue for positive regions and red for negative regions. (**C**) A view of the active site, showing details of interactions between waters and the scissile phosphate. The key hydrogen bonding interactions and zinc contacts with the RNA are shown as dashed lines. Residues that coordinate the two Zn-ions are shown in stick representation. (**D**) Substrate backbone interaction network at the active site. The view shows the participation of the D88 side chain in the water network at the active site and the amide of E87 in phosphate binding as well as the interaction of the 2′ OH of the terminal base with the scissile phosphate. In relation to Figure 1C, the perspective involves rotation about the vertical axis. These and other figures use the following color scheme: oxygen in red, nitrogen in blue, phosphate in orange, carbon in gray, Zn as gray spheres, and water as red spheres.

The crystal structure of *S. coelicolor* RNase J was solved by combining phases calculated from a molecular replacement solution with experimental phases obtained from the single wavelength anomalous dispersion signal arising from the intrinsic zinc ions. The zinc ions were not included in the search model, and their locations were corroborated by an anomalous Fourier synthesis (Supplementary Figure S3A). The structure of *S. coelicolor* RNase J is shown in Figure 1A. The enzyme shares the same structural motifs found in the *B. subtilis*, *T. thermophilus* and *Deinococcus radiodurans* homologues and other members of the family (Supplementary Figure S1) (22–24).

The asymmetric unit of the crystal encompasses a homo-dimer, and a tetramer is generated through application of crystallographic 2-fold rotation symmetry (Supplementary Figure S3B). A tetrameric quaternary structure for *S. coelicolor* RNase J is consistent with results from analytical ultracentrifugation (data not shown). The *S. coelicolor* tetramer resembles RNase J from *B. subtilis*, *T. thermophilus* and *Deinococcus radiodurans*, but unlike those enzymes, the C-terminal domain (residues 453–561) is poorly resolved in the electron density map; this domain may be folded but not in a fixed conformation with respect to the β-lactamase/β-CASP core. The loop region between residues 55 and 59 in the β-lactamase domain of *S. coelicolor* RNase J also has poorly defined electron density and may lack defined conformation.

### Uncleaved RNA is poised at the catalytic center

In the course of preparing the recombinant *S. coelicolor* RNase J, RNA originating from the *E. coli* expression host was found to co-purify with the enzyme. This RNA remained associated with RNase J during crystallisation, and interpretable density for the RNA was present in the early electron density map calculated from a molecular replacement model in the apo-form for which RNA was not included in the phase calculations (Supplementary Figure S2). Analysis of the RNA extracted from the crystals by mass spectrometry indicated the length to be eleven nucleotides (result not shown; method in Supplementary Data). The RNA was also sequenced and identified as 5′ pCGCCU, but the remaining 3′ nucleotides could not be identified unambiguously (see Materials and Methods). The 5-nucleotide sequence fitted well into the experimental electron density in the substrate-binding tunnel that encompasses the active site (Figure 1A, B), but the electron density was not clearly defined for the remaining nucleotides to the 3′ end of the experimentally identified pCGCCU sequence. The RNA is likely to be a cleavage product resulting from RNase J activity on an *E. coli* transcript.

As found for other members of the metallo-β-lactamase family, *S. coelicolor* RNase J carries two closely spaced Zn ions that are coordinated by conserved residues in the β-lactamase domain (these are annotated in Supplementary Figure S1A) (1,6–9,22–24). The RNA interacts with both the metals through the O1 and O2 oxygens of the penultimate phosphate of the 5′ terminus (Figure 1C). The 2′-OH group of the terminal furanose interacts with numerous groups, including the scissile phosphate and the His and Asp residues that coordinate the Zn; this suggests that the substrate itself may assist catalysis (Figure 1D). There is also a helix capping interaction of the E87 peptide-backbone amide with the phosphate of C2 (Figure 1D).

Perhaps the most surprising contact is a water molecule bound to both the Zn ions and close to the scissile phosphate (labeled wat1 in Figure 1C and D, Figure 3B and C). The water forms a close contact to the phosphate ester oxygen (2.7 Å). It was unexpected that the RNA would be found in the catalytic site adjacent to a water molecule that is apparently ‘activated’ by Zn binding, and yet not be cleaved. A water is present also at the active site of the complex of RNA bound to *D. radiodurans* RNase J D175A inactive mutant and is near the scissile phosphate (Figure 3C), but the water must be displaced in the wild type enzyme to avoid steric clash with D175 (Figure 3D).

**Figure 2.**
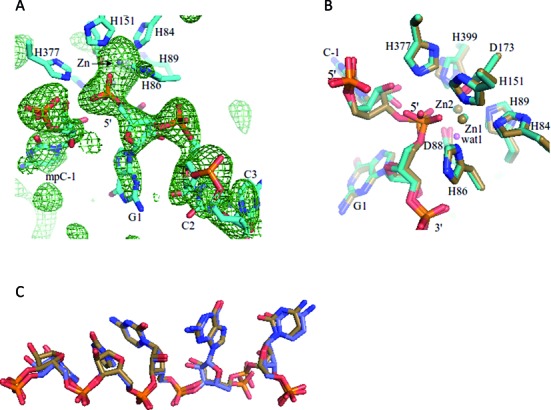
A post-cleavage complex. (**A**) A Fo–Fc map (in green mesh) using data from Mn^++^-soaked crystals and phases calculated by omitting the RNA from the refined model. The map is countered at 2.5 sigma. The refined RNA (in stick) model is superimposed onto the omit map for comparison. (**B**) A comparison of pre-cleavage RNase J and RNA complex (in sand) with the post-cleavage complex (in teal). The proposed activated water molecule (labeled ‘wat1’) from the pre-cleavage complex is pink. (**C**) A comparison of the RNA in the pre-cleavage (in sand stick representation) with post-cleavage states (in blue stick representation).

**Figure 3.**
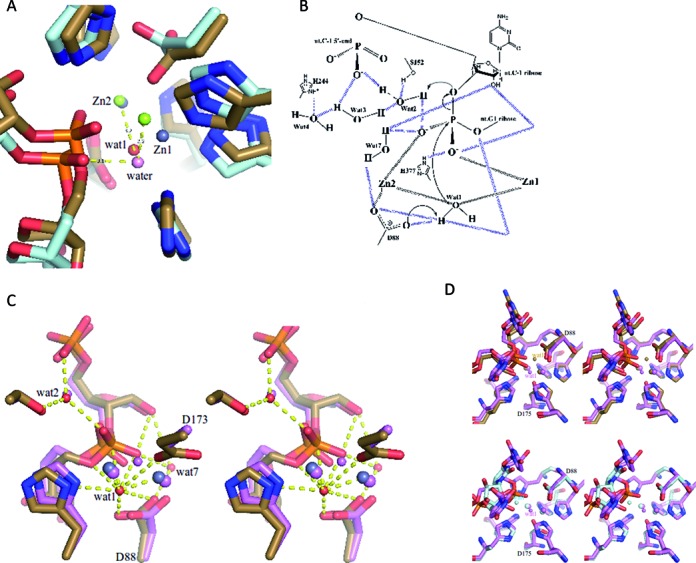
Proposed catalytic mechanism of RNase J. (**A**) A hypothetical model of the approach to the transition state. The active site, as seen in the pre-cleavage structure (sand for protein, gray spheres are for Zn ions) and attacking water (red sphere) superimposed on a model of the transition state (pale cyan for protein, green spheres are for Zn ions) in which the attacking water (pink sphere) is in-line for reaction. The model was calculated using molecular dynamics simulation. (**B**) Detailed proposed catalytic mechanism of RNase J based on the crystal structure of the *S. coelicolor* RNase J/RNA complex and the suggested mechanism for RNase Z of de la Sierra-Gallay et al. (9) and for *D. radiodurans* RNase J of Zhao et al. (7). The 5′ terminal phosphate may assist in catalysis by orienting a water chain for proton channeling. The electron transfer might involve additional waters in the network. Dashed lines indicate hydrogen bonds and metal-interactions, and the arrows indicate electron transfer. (**C**) A stereoscopic comparison of water interactions at the scissile phosphate and 5′-end. RNase J from *S.coelicolor* is in sand color, *D. radiodurans* with RNA (PDB code: 4XWW) is in pink. Zn ions are shown as larger pink and gray spheres, waters are the smaller pink and red spheres. The hydrogen bonding interactions are for the *S. coelicolor* structure. (**D**) The upper panel shows a stereoscopic comparison of active sites of RNase J from *S. coelicolor* (in sand color) and *D. radiodurans* D175A mutant in pink (4XWW) in pink; both structure have RNA bound. The lower panel shows an overlay of the mutant *D. radiodurans* with bound RNA (pink) and the wild type enzyme with UMP bound (cyan) (4XWT). The overlay shows that the activated water position seen in the mutant structure is sterically impeded by D175. The sand colored ‘Wat1’ is the proposed activated water in the S. coelicolor complex.

The reason that the *S. coelicolor* RNase J/RNA complex is stalled appears to be related to the presence of 1.33 mM EDTA in the crystallization condition, because we observe that EDTA inhibits the activity of the enzyme in this concentration range (Supplementary Figure S4). Addition of excess Mg^++^ or Mn^++^ to reaction buffer with EDTA restores activity, showing that the inhibitory effect is due to metal chelation. However, the EDTA has not removed the zinc from the active site (anomalous Fourier, Supplementary Figure S3A), so this cannot explain the loss of activity. Taken together, these results suggest that Mg^++^ is a required cofactor for the activity of RNase J. It is interesting to note that Mg^++^ is often required in cleavage assays for other members of this family, including the DNA repair enzyme Artemis and polyadenylation factor CPSF-73 (25–27). An analysis of *D. radiodurans* RNase J shows that Mg^++^ or Mn^++^ contributes to the exonuclease activity of that enzyme (7).

Close inspection of the active site shows that the scissile phosphate is not properly aligned with the potentially attacking water molecule to permit a bimolecular nucleophilic (S_N_2) attack (Figure 3A). In order for catalysis to proceed, it appears that the RNA molecule must maneuver to line up with the water. A model was developed in which the RNA conformation was adjusted so that the scissile phosphate is in-line with the activated water (Figure 3A). We suggest that this movement requires the presence of a helper ion, such as Mg^++^ or Mn^++^, as discussed further below.

### The structure of a post-cleavage complex

To investigate the role of Mg^++^/Mn^++^ as co-factor of RNase J catalysis, the co-crystals of the RNase J/RNA complex were soaked with buffer containing MnCl_2_. The structure of this soaked complex was solved by molecular replacement with a search model from the pre-cleavage RNA and RNase J complex, where RNA, Zn-ions and waters had been removed. The unbiased map confirms that the RNA has been cleaved in one of the protomers of the asymmetric unit (Figure 2A). A single nucleoside monophosphate cleavage product remains bound at the 5′ terminal recognition pocket, although in a new orientation, and a new 5′-end RNA fragment is engaged at the active site in one protomer of the dimer (Figure 2B, C).

It was not possible to identify bound Mn^++^ from the difference map or anomalous Fourier for the *S. coelicolor* enzyme. In *D. radiodurans*, Mn^++^ is bound at the dimer interface, but there is no detectable metal present at the corresponding position in the soaked crystals of *S. coelicolor* RNase J. Perhaps the metal might interact weakly or transiently with the substrate to drive the conformational change required to align the scissile phosphate with the activated water molecule.

### A proposed catalytic network

Based on the crystal structures of the *Streptomyces* RNase J/RNA complexes, a detailed mechanism for hydrolytic cleavage of the RNA can be proposed. The water coordinated to the zinc ions is activated to a hydroxyl ion as shown in the schematic of Figure 3B, which is based on the mechanism for RNase Z (10) and *D. radiodurans* RNase J (7). Residue D88 in the active site is conserved in the enzyme family (Supplementary Figure S1), and it likely acts as a general base to form the activating hydroxyl group that attacks the scissile phosphate. Substitution of the corresponding residue with alanine in *D. radiodurans* RNase J results in loss of exonuclease activity (7). The cleavage process requires protonation of the furanose 3′ oxygen at the cleavage site, and the proton most likely originates from D88 or a water molecule in the extensive hydration chain. The 5′ terminal phosphate may assist catalysis by orienting a chain of water molecules for proton channeling (Figures 1C and 3B); one implication of this substrate-assisted catalysis is that a 5′ phosphate group may favor the exo-nuclease cleavage mode (see further below). Finally, a metal such as Mg^++^ or Mn^++^ may be required to assist the movement of the scissile phosphate so that it becomes in-line with the attacking water (‘wat1’, Figure 1C and D, Figure 3B and C)). This could occur indirectly, by the metal binding at a distance to effect conformational adjustments, or through direct binding to the scissile phosphate to counter unfavorable charge distribution in the transition state. This is an inference, because there is no electron density apparent for metal in the crystal structure of the *S. coelicolor* RNase J post-cleavage complex.

### A sandwiching pocket for bases adjacent to the cleavage site

**Figure 4.**
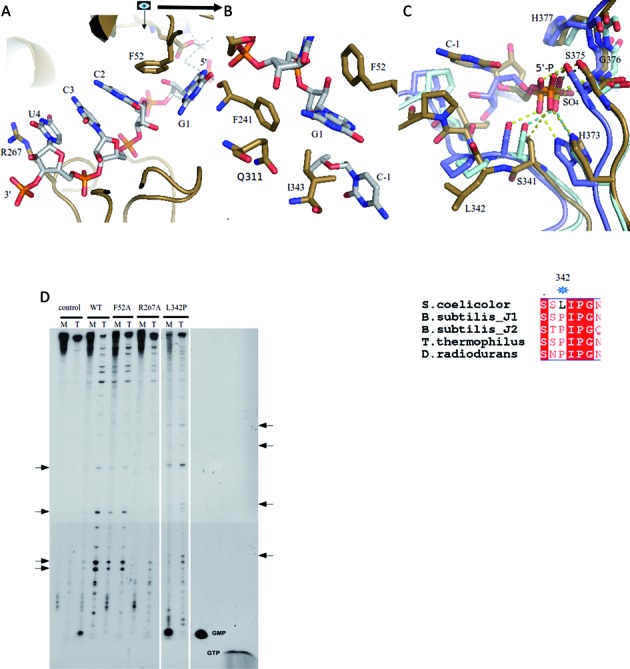
Pockets for substrate sandwiching and 5′ sensing affect exo-ribonuclease activity of *S. coelicolor* RNase J. (**A**) Inter-domain sandwiching of the substrate. The pyrimidines adjacent to the scissile phosphate bond are sandwiched between R267 from the β-CASP domain and F52 from the β-lactamase domain. (**B**) RNA G1 is engulfed in a hydrophobic pocket formed by F52, F241, I343 and the C_γ_-atom of the side chain of Q311. The scissile phosphate between C-1 and G1 is not shown for clarity. (**C**) The region around L342 is involved in 5′ sensing. Overlay of the 5′-end of *S. coelicolor* RNase J (sand) with *T. thermophilus* RNase J (pale-cyan) (PBD code: 3BK1) and *D. radiodurans* (light blue). The 5′ monophosphate group seen in *S. coelicolor* RNase J makes similar interactions to those found for an inorganic sulfate ion observed in *T. thermophilus* RNase J. Hydrogen bonds are indicated by dashed lines. The bottom panel shows a structure based sequence alignment around the L342 region. The asterisk indicates the site of substitution for the L342P mutant characterized for activity. (**D**) Cleavage of the *B. subtilis thrS* transcript with 5′-monophosphorylated (M) or 5′-triphosphorylated (T) ends, and the effects of mutations at the sandwiching (R267, F52) and 5′ sensing (L342) pockets. The standard GMP and GTP bands are indicated, and products with these mobilities are expected for the 5′-exo cleavage mode with the M and T substrates, respectively. Note that no GTP was observed with the T substrate. Arrows on the left of the figure represent products of endo- cleavage that increased with the T substrate as compared with the M substrate. Arrows on the right side of the figure indicate the positions of new endo- cleavage products observed with the L342P mutant of RNase J.

The structure shows that nucleotides 2–5 are sequestered in a pocket in which the bases are stacked and sandwiched between R267 from the β-CASP domain and F52 from the β-lactamase domain (Figure 4A). R267 makes a cation-pi interaction with base 5, and F52 makes an aromatic stack with base 3 and an edge/face interaction with G1, which is base 2. A hydrophobic pocket formed by F52, F241 and I343 engulfs base 2 (Figure 4B). Similar interactions are seen in the co-crystal structure of the inactive mutant of *D. radiodurans* RNase J with RNA (7) and in the catalytically active *T. thermophilus* RNase J in complex with 2′-O-methyl RNA (8). For the latter, two bases are sequestered in the R267/F52 pocket rather than the three seen for the *S. coelicolor* and *D. durans* enzymes, and this difference might be a consequence of the 2′-O-methyl modification.

### 5′ end sensing and the preference for endo- or exo-mode

To assay the activities of the *S. coelicolor* RNase J, we used the well-characterized *B. subtilis thrS* transcript as the substrate (4). In the activity assays used here, the ribonuclease is in excess over substrate. *S. coelicolor* RNase J exonucleolytic activity predominated when the substrate carried a 5′ monophosphate, as judged by the generation of nucleoside monophosphate product, while endonucleolytic activity predominated with the 5′-triphosphorylated substrate (Figure 4D). It is especially noteworthy that, under our assay conditions, no GTP was produced when 5′ triphosphorylated *thrS* was the substrate, showing that the exo activity is not occurring (Figure 4D). These observations suggest that *S. coelicolor* RNase J recognizes the 5′ end of the substrate and that this recognition is linked to cleavage mode and determines the prevailing enzymatic activity used for substrate degradation.

Guided by the crystal structures, we explored the impact of mutations on the preferences for endo- and exonucleolytic cleavage modes. As noted above, bases 3–5 are sandwiched between F52 and R267 (Figure 4A). Substituting either R267 or F52 with alanine (R267A, F52A) diminishes the enzyme's preference for the exo- cleavage mode with the 5′-monophosphorylated substrate, as judged by the absence of nucleoside monophosphate products in the reaction with the *thrS* substrate (Figure 4D). However, the F52A mutation has a smaller impact on the endo- cleavage mode. The R267A mutation has diminished activity for some of the endo cleavages. Therefore, the sandwiching interaction of R267 and F52 with stacked bases 3–5 appears to favor hydrolytic attack in the exo- mode, but perhaps may be less important in the endo- mode. Sandwiching of nucleotides 3–5 orients nucleotide 2 by stacking on F52 and likely aides the presentation of the scissile phosphate in permissive geometry for the exo- mode. The interaction is presumably not required for the endo- mode, and interactions elsewhere might be involved in presenting the backbone for endonucleolytic cleavage.

It is interesting to note that in the *T. thermophilus* enzyme, an inorganic sulphate and UMP were bound at the position corresponding to the 5′ terminal phosphate in both *S. coelicolor* RNase J and *D. radiodurans* (Figure 4C, upper panel). The conformation of the loop region differs between these two enzymes, and this might be influenced by the substitution of the leucine at position 342 in *S. coelicolor* RNase J by proline in the corresponding position of the *T. thermophilus* enzyme (Figure 4C, lower panel). When this substitution was introduced in the *S. coelicolor* RNase J (L342P), the enzyme became a potent exonuclease while still retaining endonuclease activity for the 5′ ppp substrate (Figure 4D). These results support the notion that 5′ end sensing has a significant impact on the preference for exo versus endo modes of activity.

## DISCUSSION

### A model for RNase J catalysis

The crystal structure presented here of *S. coelicolor* RNase J complexed with a RNA molecule has revealed a small segment of RNA that has been trapped in the active site without undergoing cleavage, despite the vicinity of a water molecule that may be activated by the two zinc ions of the catalytic site. The structure reveals that the 2′ OH of the sugar adjacent to the scissile phosphate forms a hydrogen bond (3.0 Å) with the phosphate O2 oxygen, suggesting a mechanism involving substrate-assisted catalysis (Figure 1C and D). We propose a mechanistic model based on the structural features in which a network of water molecules facilitates the acid-base chemistry required for hydrolytic cleavage of the RNA substrate (Figure 3B). We also suggest a critical role of a metal co-factor that aids the movement of the scissile phosphate into a position for hydrolytic attack. Consistent with the proposal that metal is required to facilitate hydrolytic cleavage by RNase J, the structure of Mn^++^-ion soaked crystals show that the trapped RNA fragment has undergone exo-cleavage on the phosphodiester bond of C(-1) nucleotide (Figure 2). Comparing the pre- and post- cleavage crystal structures reveals that the activating water molecule (‘wat1’ in Figure 1C and D, Figure 3B and C) seen in the former is likely to be absent in the latter (with the caveat that it is a lower resolution structure). These findings account for the observation that Mg^++^ is often required in cleavage assays for other members of this family (21,25–28). Based on the results obtained here, we suggest that the metal is a facilitating co-factor for the enzymes in this family.

A sulfate ion is found in the catalytic site of the *T. thermophilus* RNase J (9) and in human glyoxalase (29) at the position corresponding to the RNA in the structures described here and in *D. radiodurans*, indicating that substrate binding by these enzymes might be similar. The RNA-zinc interactions resemble those observed in the co-crystal of RNase Z with tRNA (Supplementary Figure S5A, S5B) (28); however, the tRNA substrate in that complex is protected by 2′-O-methyl groups at the last two 3′ bases, and these modifications result in some small changes in the backbone geometry at the active site. The structure of *T. thermophilus* RNase J in complex with 2′-O-methyl-protected RNA revealed binding of the nucleic acid near the active site, but due to the modifications it is not in an orientation that mimics the cleavage state geometry (8) (Supplementary Figure S5C, S5D).

Modeling suggests that the RNA phosphate must move to become in-line with the attacking water (Figure 3A). This movement is likely restrained by the conformation of the RNA on either side of the scissile bond. Immediately adjacent to the scissile phosphate, a guanine contacts F52 from the β-lactamase domain and F241 from the β-CASP domain; F52 in turn stacks on the adjacent pyrimidine that is part of a stack of three consecutive pyrimidines (residues 2–4 from the scissile phosphate), and these are sandwiched by R267 from the β-CASP domain (Figure 4A, B). Adjustment of the conformation of the scissile phosphate may require accommodating movements of these bases (Figure 2C). The substrate-enzyme complex is therefore likely to store mechanical energy in this stalled conformation, so that cleavage is coupled with translocation of the RNA to the next cleavage site. Any mechanical strain associated with optimizing the stacking is relaxed following cleavage, and this is likely to incrementally move the substrate along, so that the bases in the sandwich shuffle and the new penultimate base is stacked against F52 for optimal presentation of the phosphate backbone. Thus, we envisage a ‘spring loaded’ model in which the cleavage of the substrate releases stored mechanical energy that can be used to move the substrate along to the next site of cleavage. A similar spring-loaded mechanism has been proposed to explain the processive behavior of the yeast hydrolytic ribonuclease component of the exosome, Rrp44 (30). In the RNase J family, both R267 and F52 residues are conserved, suggesting that this sandwiching interaction might also occur in the other members of this enzyme group. One interesting exception of the conservation of those two residues is *B. subtilis* RNase J2, potentially explaining why that protein does not have the same exonuclease activity as J1.

### Conformational switching and RNA binding

**Figure 5.**
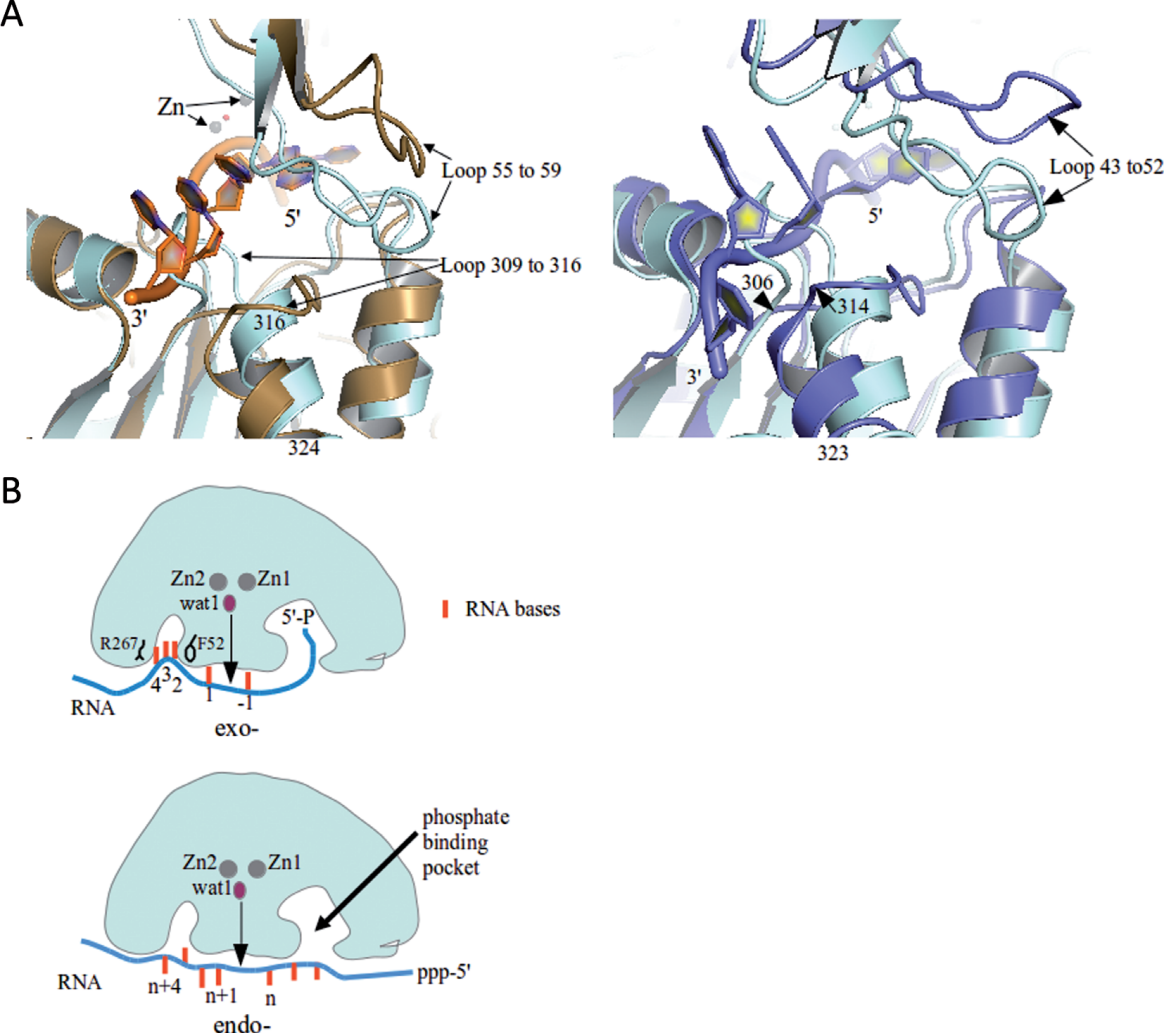
Conformational adjustments in RNase J with RNA binding and the substrate path for the exo- and endo-cleavage modes. (**A**) The left panel presents a structural overlay of the RNA-bound *S. coelicolor* RNase J (sand) with the apo-form of *Thermus thermophilus* RNase J (cyan) (PDB code: 3BK1). Loop movements may accommodate the RNA molecule (backbone in orange and base shown as filled rings). The panel on the right compares the *T. thermophilus* RNase J apo-form (cyan) and RNA-bound form (blue), where the RNA bears 2′-O-methyl modifications. (**B**) Proposed substrate interactions for exo- and endo- cleavage modes. Sandwiching of the bases between R267 and F52 and 5′ end sensing are proposed to favor the exo- mode, perhaps through a ‘spring-loaded’ mechanism. In the endo mode, the 5′ binding pocket is potentially left unoccupied. Endo cleavages may involve tetramer dissociation and re-association on the substrate and potentially some translocation along tunnels (see Supplementary Figure S5E for possible pathways for the tunnels).

RNA binding is likely to be accompanied by a structural switch in the channel from a closed to an open state, judging from comparison of the apo-form of *T. thermophilus* RNase J with the RNA bound *S. coelicolor* RNase J. In accommodating the RNA, a loop composed of residues 309–316 moves to form a tight-fitting substrate binding tunnel. Opening and accommodation of RNA substrate is also associated with the axial translational of the α-helix made from residues 316–324 (Figure 5A, left panel). Movement of the 309–316 loop is correlated with a corresponding movement of the loop 55 to 59 which has high B-factors between 80 and 100 Å^2^ in the RNase J structure. The movement is also seen in comparing the *T. thermophilus* RNase J in apo-form and bound to 2′-O-methylated RNA (Figure 5A, right panel), and in the structure of *D. radiodurans* inactive mutant in complex with RNA (not shown). Both loop regions are highly conserved, indicating that the loop conformational change with substrate interaction may be common in the family for RNA engagement.

### 5′ sensing and the switch between exo and endo modes

Earlier studies have suggested a structural rationale for the impact of the 5′ end of RNA substrates on RNase J cleavage mode (2–4). In the case of RNA substrates containing a 5′ triphosphosphate, the terminal phosphate is proposed to bind in the phosphate-binding pocket in such a way that brings the scissile phosphate out of phase with the active site. Our structural results for *S. coelicolor* RNase J suggest that the 5′ monophosphate helps to orient the penultimate sugar to interact with the scissile phosphate to potentially provide substrate-assisted catalysis (Figure 3). Modeling a triphosphate group in the pocket suggests that the alpha-phosphate may not be able to provide the same orientation due to conformational changes to accommodate the gamma and beta phosphate (not shown). Thus recognition of the 5′ end may help to organize the active site to define the exo cleavage mode.

It has also been proposed earlier that the switch from exo to endo ribonuclease mode might require the subunits of the tetramer to dissociate transiently and reform on an internal site in the single stranded region of the substrate. Our mutagenesis results suggest a role of the conserved sandwiching pocket in this switch. The stacking of bases in the pocket might influence the tetramer/dimer equilibrium. For substrates with a triphosphate on the 5′ end, the substrate may not engage in the pocket, perhaps due to energetic barriers, and the bases may not stack in the sandwiching pocket (Figure 5B). It is also possible that the RNA might glide along if it is not engaged in the pocket, and would then be cut in an endo- mode; a similar mechanism has been proposed for *Mycobacterium tuberculosis* RNase J (31). It is envisaged that the RNA can occupy tunnels in the endo- mode of cleavage (Supplementary Figure S5E). Structural rearrangement of the different domains would be required to accommodate RNA substrates in the endo- mode. As the tunnel is closed, a large domain movement likely leads to formation of an open channel around the active site in order to accommodate single stranded RNA.

## Supplementary Material

SUPPLEMENTARY DATA
