# Supplementary material for: Linkage of catalysis and 5′ end recognition in ribonuclease RNase J
Source: Nucleic Acids Res. 2015 Aug 7;43(16):8066–76. doi: 10.1093/nar/gkv732 (PMC4652760; doi:10.1093/nar/gkv732)
Supplement: SUPPLEMENTARY DATA [file supp_43_16_8066__index.html]

Linkage of catalysis and 5′ end recognition in ribonuclease RNase J — SUPPLEMENTARY DATA 

# Linkage of catalysis and 5′ end recognition in ribonuclease RNase J

## SUPPLEMENTARY DATA

- SUPPLEMENTARY DATA
- SUPPLEMENTARY DATA
- SUPPLEMENTARY DATA
- SUPPLEMENTARY DATA
- SUPPLEMENTARY DATA
- SUPPLEMENTARY DATA
- SUPPLEMENTARY DATA
- SUPPLEMENTARY DATA
