# Supplementary material for: Linkage of catalysis and 5′ end recognition in ribonuclease RNase J
Source: Nucleic Acids Res. 2015 Aug 7;43(16):8066–76. doi: 10.1093/nar/gkv732 (PMC4652760; doi:10.1093/nar/gkv732)
Supplement: SUPPLEMENTARY DATA [file supp_gkv732_nar-01315-z-2015-File010.docx]

**SUPPLEMENTARY DATA**

***Modelling the transition state intermediate of RNase J/RNA***

The P-O2 and P-O1 bound in the second phosphate group at the 5'-end of an RNA substrate was manually linearized to present a transition intermediate configuration of the phosphate. The entire RNA fragment was moved along and parallel to the plane of Zn1-Zn2-Water to allow the second phosphate group of the RNA 5'-end to line up with the attacking water molecule. The coordinates were refined in PHENIX using a weight value of zero in simulation annealing and molecular dynamics.

***Assay of RNase J activity***

Chemically synthesized 33 nucleotide long RNAs with either 5'-monophosphate ends or 5'-OH protected ends were used as substrates in the RNase J activity assay. Ten µl assay mixtures contained 1x buffer A, B, C or D (Buffer A containing 100 mM NaCl, 50 mM Tris-HCl, 10 mM MgCl_2_ [or 2.5 mM MgCl_2_], 1 mM DTT (dithiolthreitol), pH 7.9; Buffer B containing 100 mM NaCl, 50 mM Tris-HCl, 1 mM DTT, 2 mM EDTA [or 2.5 mM EDTA], pH7.9; Buffer C containing 100 mM NaCl, 50 mM Tris-HCl, 10 mM MgCl_2_, 1 mM DTT, 2 mM EDTA, pH 7.9, Buffer D containing 100 mM NaCl, 50 mM Tris-HCl, 5 mM MgCl_2_, 1 mM DTT, 5 mM EDTA, pH 7.9,) with 52.7 pM RNase J wild type or mutant L342P and 2 pM substrate. Reactions were incubated at 37ºC and terminated by addition of 10 µl of 0.5 mg/ml proteinase K in a buffer containing 100 mM Tris-HCl pH 7.5, 20 mM EDTA, 150 mM NaCl, 1% SDS followed by incubation at 55ºC for 30 minutes. The product were then separated by 4 % and 20 % Urea-polyacrylamide gel. Gels were stained with Sybr-Gold (Life Technologies) and viewed under UV.

The sequence of the 33-mer RNA used for activity tests is

5'OH-AGUAGCCUUGCUAUUUCAGUGGCGAAUGAUGAU.

***RNA mass spectrometry analysis***

RNA samples extracted from crystals and protein used for crystallisation were adsorbed to C18Ziptip (Millipore) and washed with 5% acetic acid to remove salt. Elution to the MALDI analysis plate was with trihydroxy-acetophenone (THAP) matrix solution, comprising 0.1M 2,4,6-THAP, 0.05M 2,3,4-THAP and 0.075M di-ammonium citrate (all Sigma-Aldrich) in 3:5 (v/v) acetonitrile/water. Samples were allowed to dry in air. Analysis was performed on a Water MALDI micro MX mass spectrometer operating in linear and reflectron mode according to manufacturer specifications (1).

SUPPLEMENTARY REFERENCES

1. Gruis-Sovulj,I., Ludemann,H.C., Hillenkamp,F., Weygand-Durasevic,I., Kucan,Z and Jasna Peter-Katalinic,J. (1997) Matrix-assisted laser desorption/ionisation mass spectrometry of transfer ribonucleic acids isolated from yeast. *Nucleic Acids Res.*, **25(9)**, 1859-1861.

**SUPPLEMENTARY FIGURE LEGENDS**

**Supplementary Figure S1** Structure based sequence alignment of RNase J homologues (http://esprit.ibcp.fr/ESPript/ESPript). The numbering scheme is according to *S. coelicolor.* Colour bars above the sequences indicate domain boundaries: sand colour is for the β-lactamase domain, cyan for the β-CASP domain, and orange for the C-terminal domain. Black arrows represent β-strands, black springs for helices, and T is for turns. Conserved residues are in red highlighted in white font, similar residues are in red font and framed with blue lines. Residues indicated with a blue star are involved in zinc coordination or catalysis. The sequences are underlined for motifs I, II, III and IV for the β-lactamase domain, and A,B, V/C for the β-CASP domain using the convention according to references 22-24.

**Supplementary Figure S2** Unbiased maps showing RNA in the crystal. (A) Initial Fo-Fc map from molecular replacement using a model in which there was neither RNA nor active site zinc. The final refined RNA structure is superimposed on this difference map. The difference map is shown in grey mesh at 2.7 sigma level, and the RNA model is in stick representation. (B) A Fo-Fc omit map of a single nucleotide after removing the nucleotide from the model followed by refinement with simulated annealing. The map is shown at 2.7 sigma level. (C) The Fo-Fc omit map of the entire RNA fragment after phasing with simulated annealing, shown at 2.7 sigma level.

**Supplementary Figure S3** (A) Anomalous Fourier showing location of zinc ions. The map was calculated using anomalous coefficients from data collected at the Zn peak and phases calculated from the molecular replacement model in which the zinc ions were removed. (B) Cartoon representation of tetrameric *Streptomyces coelicolor* RNase J. Bound RNA is shown in ribbon representation and in filled stick form (pink). The domains are color-coded in one of the subunits, as indicated by the annotated bar beneath the cartoon. The other subunits of the tetramer are shown in different shades of grey. The C-terminal domain could be seen in the experimental map, and its location is depicted as oval-shape cartoons labelled ”Ct”. The left and right panels are related by a 90º degree rotation along the viewing axis.

**Supplementary Figure S4** Effects of EDTA and Mg^++^ on RNA cleavage by RNase J. (A) 20% Urea-acrylamide gel analyses of the cleavage of a 5'-OH-33-mer RNA, with RNase J mutant L342P. EDTA inhibits cleavage, while Mg^++^ supports the activity. The inhibitory effect of EDTA is overcome by adding excess Mg^++^, suggesting that the EDTA effect is due to sequesting the metal co-factor. EDTA is unlikely to be inhibiting by removing the zinc from the enzyme, since the pre-cleavage state crystals were prepared in the presence of 2 mM EDTA, but zinc is still present at the active site. (B) 4% Urea-acrylamide gel analyses of the cleavage of *E. coli* 16S and 23S with wild type RNase J in the presence of EDTA and Mg^++^.

**Supplementary Figure S5** Comparison of the active sites for *S. coelicolor* RNase J and its homologs. (A) Structural comparison between *S. coelicolor* RNase J (in sand)/RNA (in orange) complex and RNase Z (in light purple) /tRNA (in blue) complex. (B) Zoom into the active sites for *S. coelicolor* RNase J and RNase Z. The attacking water in *S. coelicolor* RNase J/RNA complex is in red, and hydrogen bonds are shown as dashed-lines. Amino acids are labelled in *S. coelicolor* RNase J/RNA complex structure. Hydrogen bonds in the RNase Z/tRNA are in solid-lines. (C) The configuration of the zinc coordination site *S. coelicolor* RNase J is shown in sand, and the *Thermus thermophilus* enzyme with modified RNA; the chains are in green, pink, grey and cyan (PDB code 3IEM). (D) Comparison of the RNA in the active site of *S. coelicolor* (sand) and *Thermus thermophilus* with 2'-O methyl modified RNA (light blue) (PDB code 3T3N). (E) Four-way tunnels that may accommodate single-stranded regions at the active sites in the endo cleavage mode. The suggested binding channels are indicated by dashed orange lines.

Table S1 Crystallographic statistics

| **Data collection and procession** | | |
| --- | --- | --- |
|  | Pre-cleavage  (Zn-peak) | post-cleavage (Mn soaked) |
| Space group | P4_3_22 | P4_3_22 |
| Cell dimensions |  |  |
| a, b, c (Å) | 186.19, 186.19, 113.38 | 184.13, 184.13, 112.68 |
| α, β, γ, (º) | 90.0, 90.0, 90.0 | 90.0, 90.0, 90.0 |
| Wavelength (Å) | 1.2829 | 0.9174 |
| Resolution (Å) | 29.5 - 2.3 | 37.8 – 2.7 |
| R_merg_(%)^#,1^ | 5.1 (49.4) | 13.9 (76.6) |
| CC1/2 | 0.999 (0.757) | 0.993 (0.476) |
| R_anom_^#,2^ | 2.28 (35.3) | - |
| <I / σI>^#^ | 10.6 (2.5) | 9.7 (2.5) |
| No. of reflections^#^ | 727680 (25777) | 518053 (41645) |
| No. of reflections (unique) | 88741 (3391) | 39850 (2892) |
| Redundancy^#^ | 8.2 (7.6) | 13.0 (14.4) |
| Completeness (%)^#^ | 99.3 (97.9) | 99.8 (99.5) |
| Anomalous Completeness^#^ | 96.1 (88.5) | 99.8 (99.0) |
| Anomalous multiplicity^#^ | 4.3 (2.7) | 6.9 (7.5) |
| delAnomalous Correlation  between half-sets (%)^#^ | 11.5 (0.7) | 21.6 (0.005) |
| No. protomer /  asymmetric unit | 2 | 2 |
| **Phasing** | | |
| Methods | SAD + molecular replacement | molecular replacement |
| Search model  (PDB code) | 3BK1 | pre-cleavage model (removal of RNA, Zn and waters) |
| Heavy Atom Sites | 4 Zn-sites |  |
| FOM | 0.309 |  |
| **Refinement** | | |
| Resolution (Å) | 29.5 – 2.3 | 30 – 2.8 |
| No. reflections (unique) | 88551 | 47608 |
| R_work_(%) / R_free_(%)^4^ | 13.28 / 16.29 | 15.63 / 20.83 |
| Structural regions | 1 - 452 | 1 - 452 |
| *No. Atoms* | | |
| Total | 9157 | 7232 |
| Protein | 8118 | 8118 |
| RNA | 254 | 255 |
| Water | 781 | 72 |
| Metal ions (Zn) | 4 | 4 |
| *B-factor* | | |
| Overall | 51.1 | 50.5 |
| Protein | 68.4 | 54.5 |
| RNA | 80.0 | 68.5 |
| Water | 42.2 | 61.3 |
| Metal ions | 52.1 | 58.1 |
| *r.m.s deviation* | | |
| Bond length (Å) | 0.008 | 0.008 |
| Bond angle (º) | 1.242 | 1.135 |
| PDB code | 5A0T | 5A0V |

^#.^ Values in parentheses are for highest-resolution shell. Data for RNase J structure were collected from a single crystal.

^1.^ R_merge_=∑_h_ ∑_i_ | I_i_ (h) - <I(h)> | / | ∑_h_ N <I(h)>, were I_i_ (h) is the i^th^ measurement and <I(h)> is the weighted mean of all measurements (N) of I(h).

^2.^ Anomalous signal containing flections only.

Where:

I_j_ = the intensity of the jth observation of reflection i

<I_i_> = the mean of the intensities of all observations of reflection i

N_i_ = the redundancy (the number of times reflection i has been measured).

∑_i_ is taken over all reflections

∑_j_  is taken over all observations of each reflection.

^4.^ R_free_ = the R-factor obtained from 5% of the reflections not included in the refinement.
