# Supplementary figures and images for: Linkage of catalysis and 5′ end recognition in ribonuclease RNase J
Source: Nucleic Acids Res. 2015 Aug 7;43(16):8066–76. doi: 10.1093/nar/gkv732 (PMC4652760; doi:10.1093/nar/gkv732)

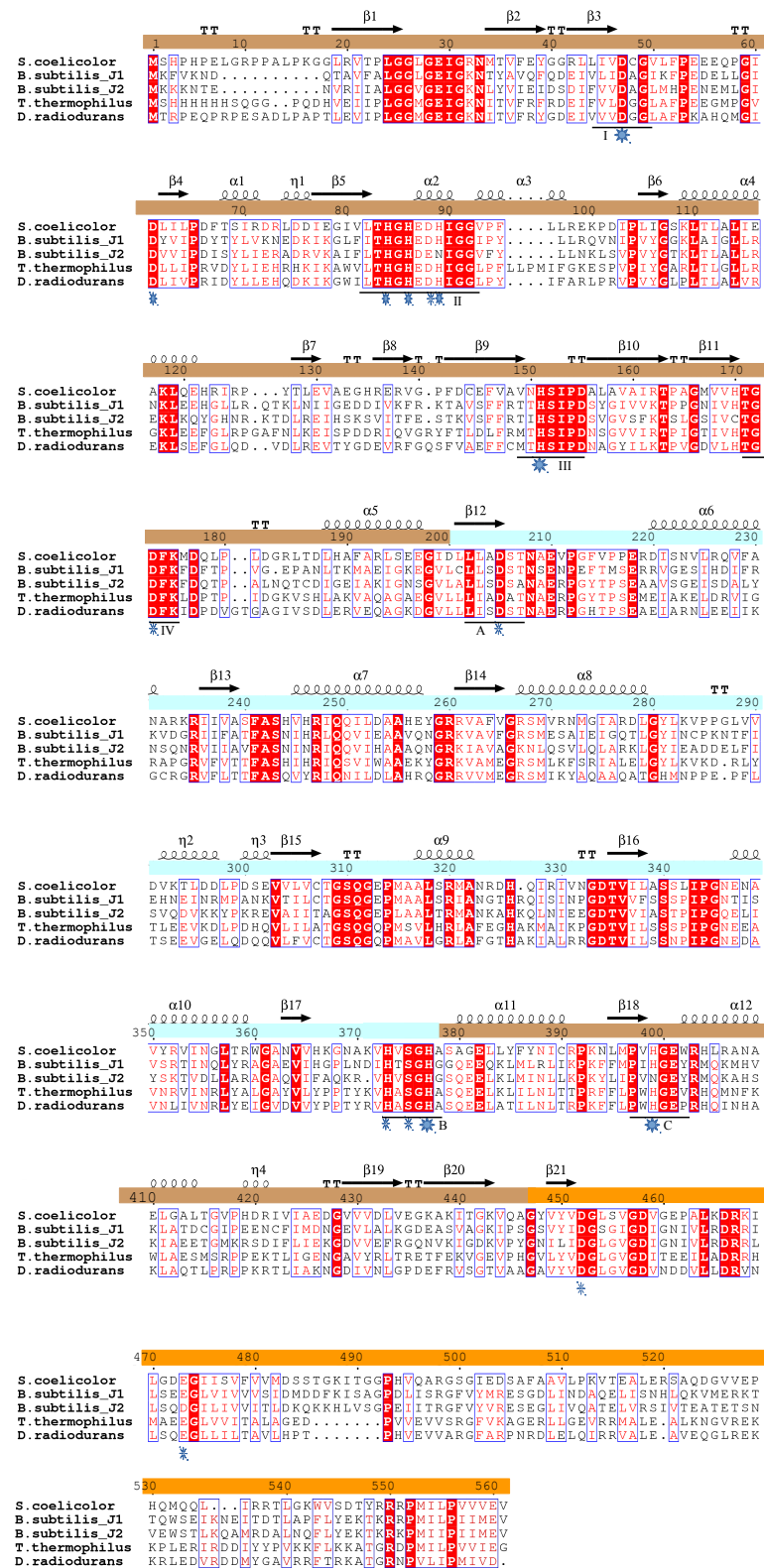

Supplement: SUPPLEMENTARY DATA [file supp_gkv732_nar-01315-z-2015-File011.pdf]

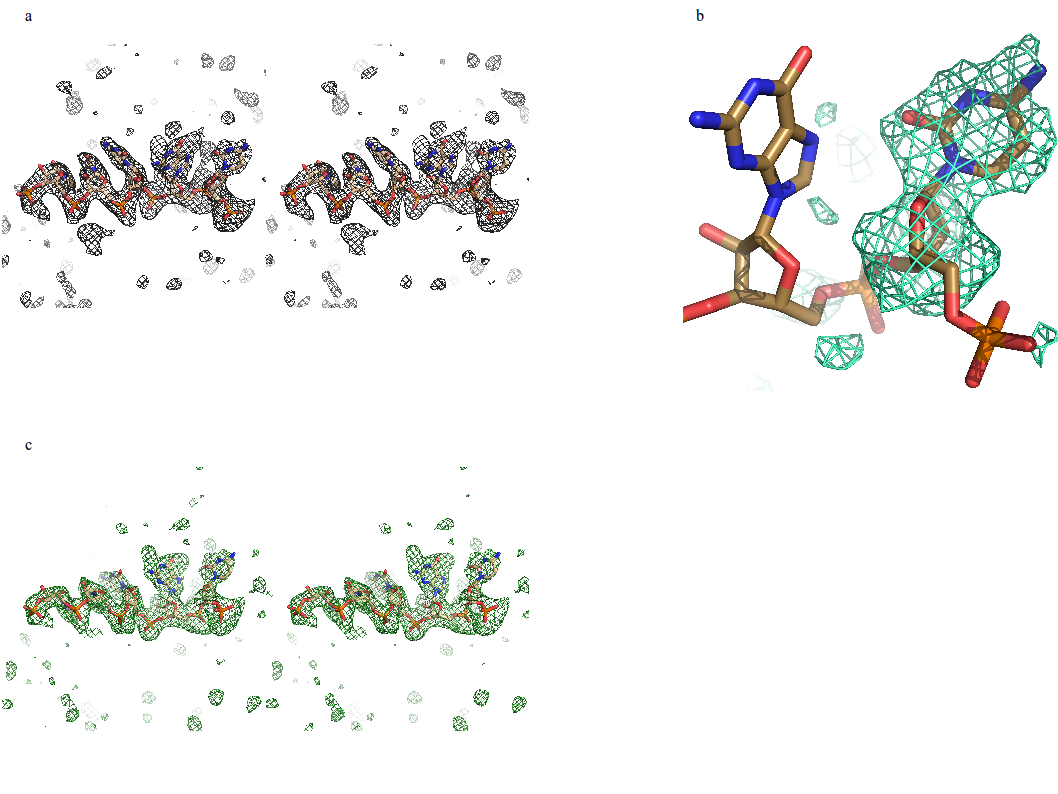

Supplement: SUPPLEMENTARY DATA [file supp_gkv732_nar-01315-z-2015-File012.tif]

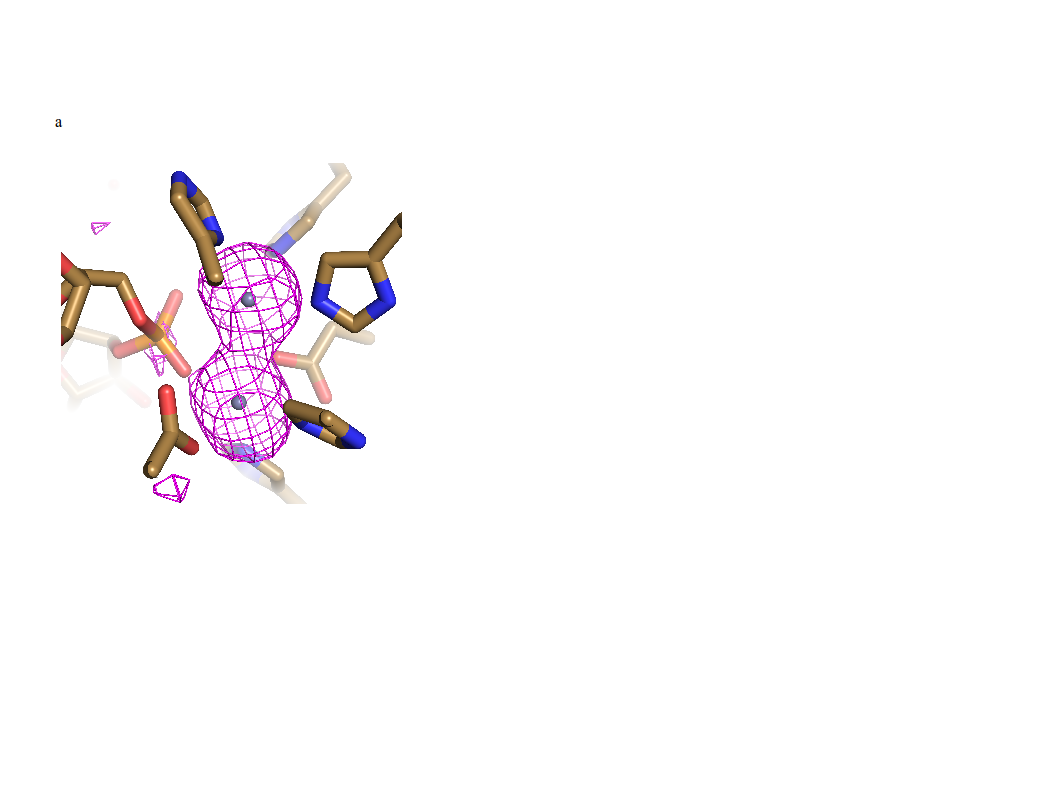

Supplement: SUPPLEMENTARY DATA [file supp_gkv732_nar-01315-z-2015-File013.tif]

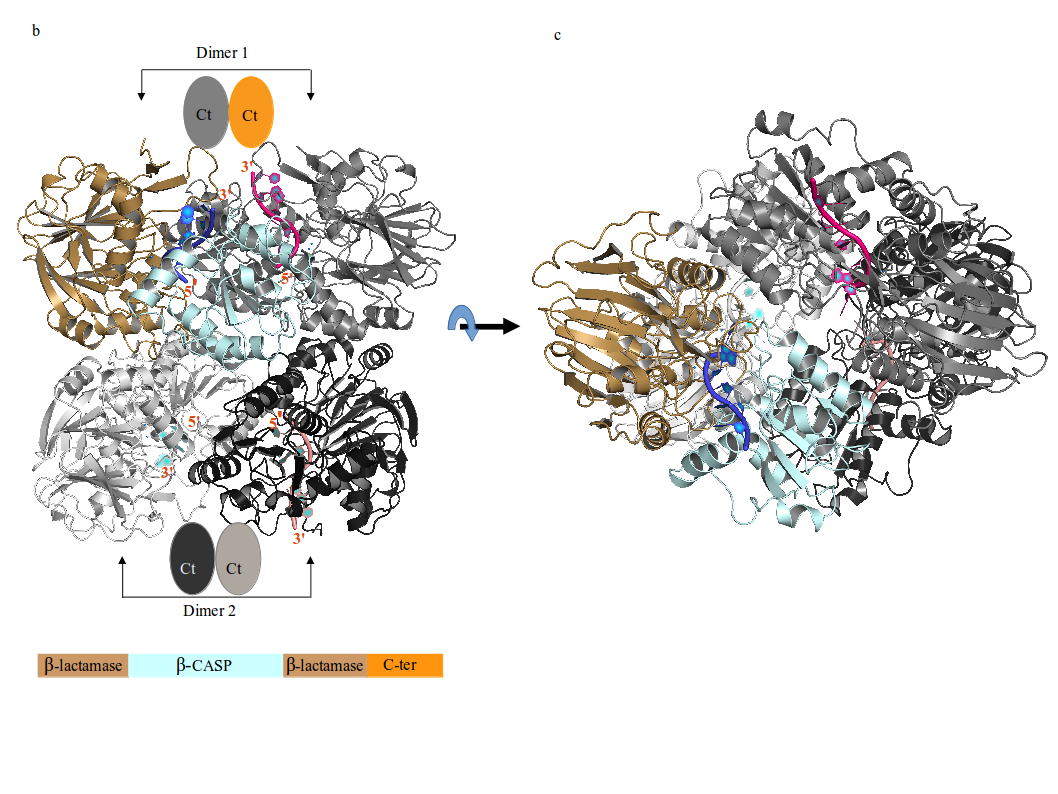

Supplement: SUPPLEMENTARY DATA [file supp_gkv732_nar-01315-z-2015-File014.tif]

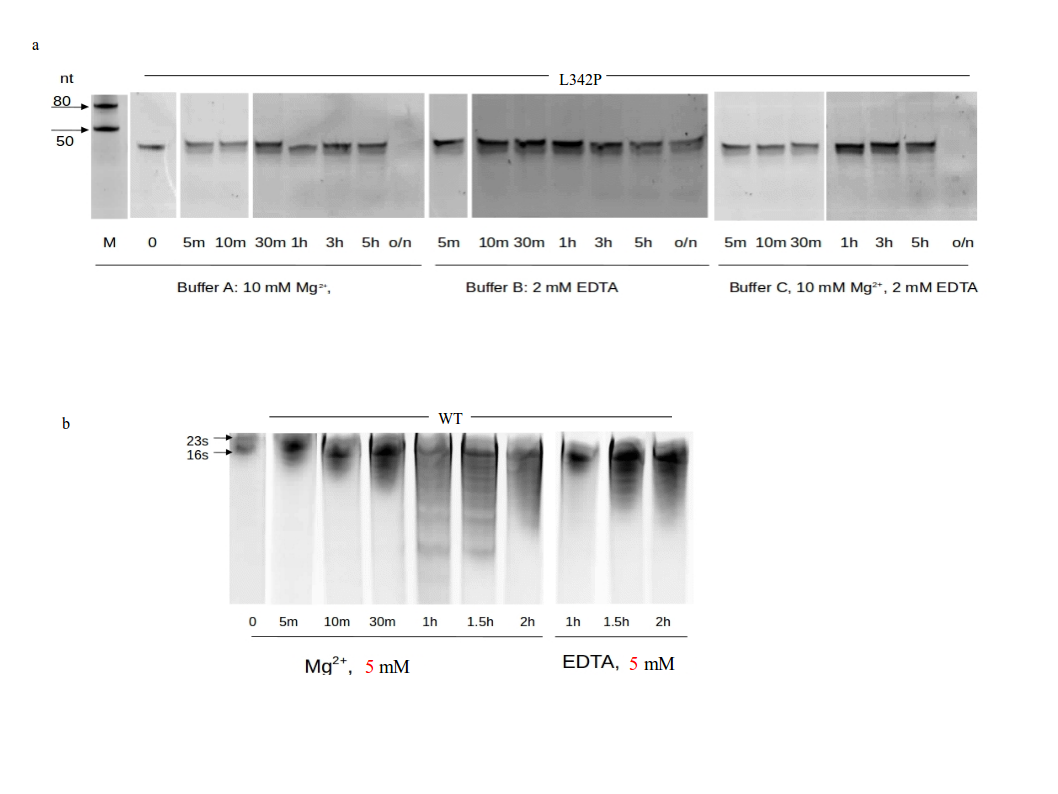

Supplement: SUPPLEMENTARY DATA [file supp_gkv732_nar-01315-z-2015-File015.tif]

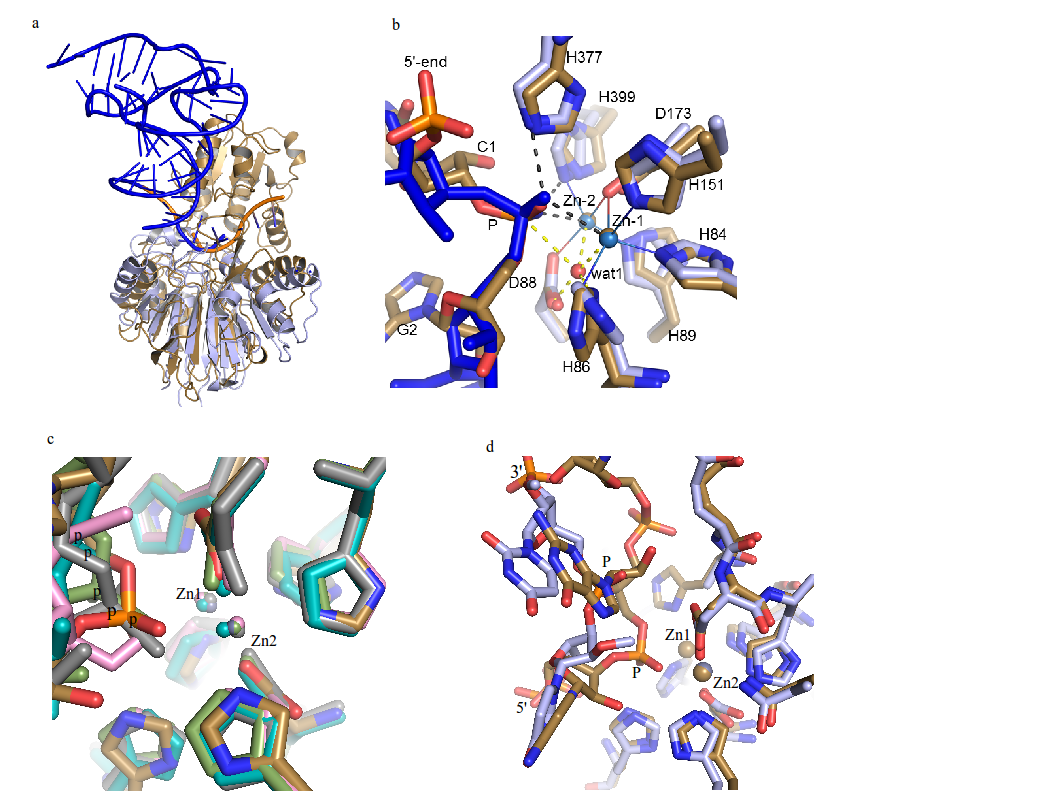

Supplement: SUPPLEMENTARY DATA [file supp_gkv732_nar-01315-z-2015-File016.tif]

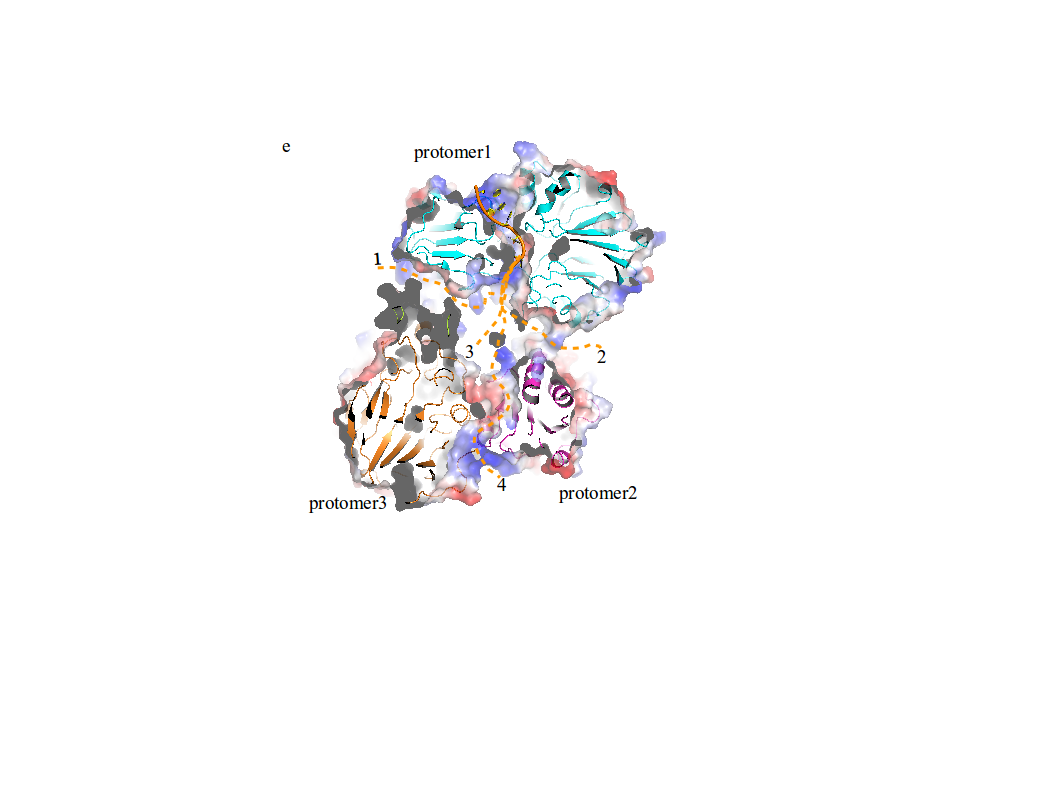

Supplement: SUPPLEMENTARY DATA [file supp_gkv732_nar-01315-z-2015-File017.tif]
